# Supplementary material for: Reliable CRISPR/Cas9 Genome Engineering in Caenorhabditis elegans Using a Single Efficient sgRNA and an Easily Recognizable Phenotype
Source: G3 (Bethesda). 2017 Mar 7;7(5):1429–37. doi: 10.1534/g3.117.040824 (PMC5427500; doi:10.1534/g3.117.040824)
Supplement: Supplementary file 1 [file 1429FileS1.pdf]

Supplementary information for

Reliable CRISPR/Cas9 genome engineering in *Caenorhabditis elegans* using a single efficient sgRNA and an easily recognizable phenotype

Sonia El Mouridi, Claire Lecroisey, Philippe Tardy, Marine Mercier,

Alice Leclercq-Blondel, Nora Zariohi, Thomas Boulin[§]

Institut NeuroMyoGène

Univ Lyon, Université Claude Bernard Lyon 1

CNRS UMR 5310, INSERM U1217

8 Rue Raphaël Dubois

69100, Villeurbanne, France

[§] Corresponding author: thomas.boulin@univ-lyon1.fr

Figure 2 Supplement 1

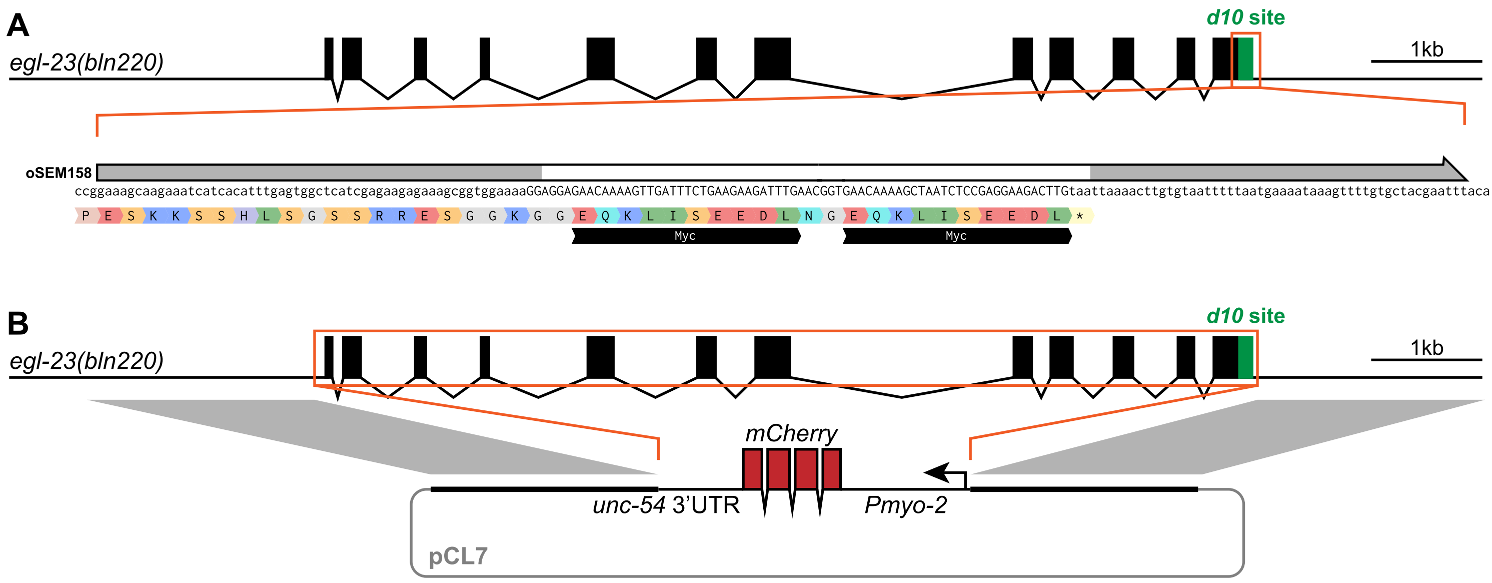

Figure 2 Supplement 1

A Insertion of a 2xMyc tag into the *egl-23* locus using a single-strand DNA template. Correspondence of homology regions between the ssON repair template and genomic locus is indicated in gray. The sequence of the resulting fusion protein is indicated below the DNA sequence with single-letter amino acid code. Black bars labeled "Myc" indicate the position of the *myc* tag sequences.

B Deletion and replacement of the *egl-23a* locus by a *Pmyo-2::mCherry* reporter transgene. Correspondence of homology regions between the plasmid repair template (pCL7) and genomic locus is indicated in gray. The *Pmyo-2::mCherry::unc-54 3'UTR* transgene is inserted in the reverse orientation relative to the *egl-23* gene.

Figure 3 Supplement 1

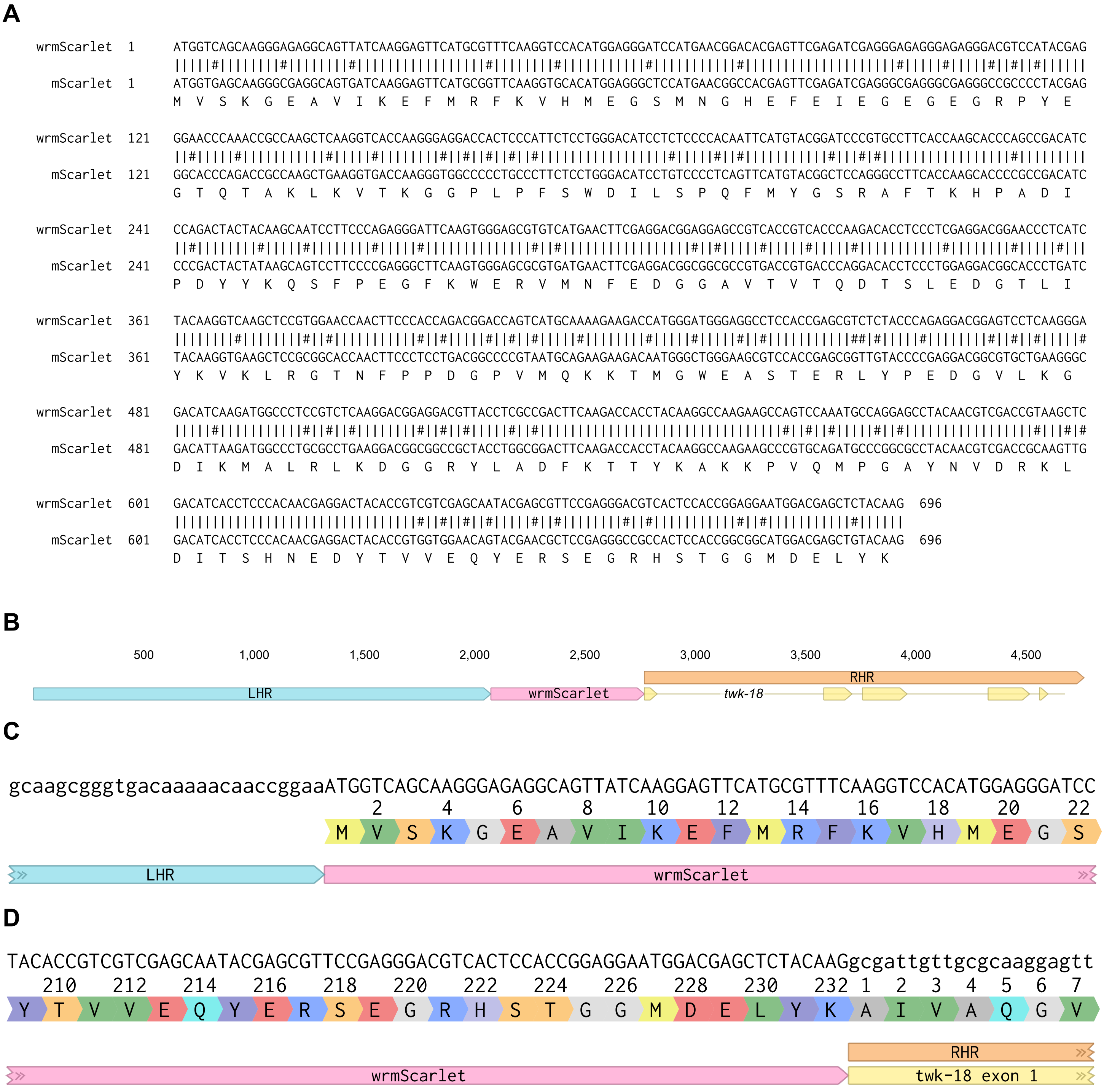

Figure 3 Supplement 1

A Codon-optimized *wrmScarlet* vs *mScarlet* sequence alignement.
B Schematic representation of the pSEM87 *wrmScarlet::twk-18 repair template*. LHR and RHR indicate left and right homology regions, respectively. The five first exons of *twk-18* present in the RHR are indicated in yellow. Scale bar in base pairs.
C 5' junction of *wrmScarlet* to *twk-18*. The sequence of the resulting fusion protein is indicated below the DNA sequence with single-letter amino acid code and corresponding amino acid positions.
D 3' junction of *wrmScarlet* to *twk-18*. The sequence of the resulting fusion protein is indicated below the DNA sequence with single-letter amino acid code and corresponding amino acid positions.

Supplementary Tables

Supplementary Table 1: Prevalence of GNGG and GGNGG protospacers in and close to exons of two-pore domain potassium channel genes.

Gene	NGG	GNGG	GGNGG	GNGG/NGG	GGNGG/NGG
egl-23	226	59	23	0.26	0.10
sup-9	115	27	10	0.23	0.09
unc-58	181	33	9	0.18	0.05
twk-3	85	8	2	0.09	0.02
twk-6	70	10	2	0.14	0.03
twk-8	70	10	2	0.14	0.03
twk-12	134	26	6	0.19	0.04
twk-13	190	49	19	0.26	0.10
twk-14	122	23	8	0.19	0.07
twk-16	146	34	10	0.23	0.07
twk-17	185	40	13	0.22	0.07
twk-18	121	18	6	0.15	0.05
twk-20	108	17	5	0.16	0.05
twk-23	118	29	7	0.25	0.06
twk-24	102	25	5	0.25	0.05
twk-25	166	25	6	0.15	0.04
twk-28	132	22	5	0.17	0.04
twk-30	139	25	7	0.18	0.05
twk-32	148	33	8	0.22	0.05
twk-40	148	33	8	0.22	0.05
twk-44	195	43	6	0.22	0.03
twk-46	132	31	5	0.23	0.04
**Average ratios**				**0.20**	**0.05**

Supplementary Table 2: Strain list

Strain	Genotype	Description
JIP1141-2	*tag-68(bln209) I*<br>*tag-68(bln212) I*	Insertion of *d10* site in 5' of *tag-68*
JIP1155-7	*sup-9(bln224) II*<br>*sup-9(bln225) II*<br>*sup-9(bln226) II*	Insertion of *d10* site in 5' of *sup-9*
JIP1127, JIP1129	*egl-23(bln172) IV*<br>*egl-23(bln179) IV*	Insertion of *d10* site in 5' of *egl-23b*
JIP1149-50	*egl-23(bln219) IV*<br>*egl-23(bln220) IV*	Insertion of *d10* site in 3' of *egl-23*
JIP1143	*twk-18(bln213) X*	Insertion of *d10* site in 5' of *twk-18*
JIP1152	*tag-68(bln222) I*	Insertion of TagRFP-T in *tag-68* at 5'
JIP1224-6	*sup-9(bln236) II*<br>*sup-9(bln237) II*<br>*sup-9(bln238) II*	Insertion of TagRFP-T in *sup-9* at 5'
JIP1368-70	*twk-40(bln282) III*<br>*twk-40(bln283) III*<br>*twk-40(bln284) III*	Insertion of TagRFP-T::ZF1 in *twk-40* at 3'
JIP1171-5	*egl-23(bln227) IV*<br>*egl-23(bln228) IV*<br>*egl-23(bln229) IV*<br>*egl-23(bln230) IV*<br>*egl-23(bln231) IV*	Insertion of TagRFP-T in *egl-23* at 3'
JIP1221-3	*egl-23(bln233) IV*<br>*egl-23(bln234) IV*<br>*egl-23(bln235) IV*	Insertion of SL2::TagRFP-T in *egl-23* at 3'
JIP1336	*egl-23(bln277) IV*	Insertion of TagBFP in *egl-23* at 3'
JIP1448-50	*egl-23(bln309) IV*<br>*egl-23(bln310) IV*<br>*egl-23(bln311) IV*	Insertion of wrmScarlet in *egl-23* at 3'
JIP1233-5	*twk-18(bln246) X*<br>*twk-18(bln247) X*<br>*twk-18(bln248) X*	Insertion of TagRFP-T in *twk-18* at 5'
JIP1236, JIP1251-2	*twk-18(bln249) X*<br>*twk-18(bln250) X*<br>*twk-18(bln251) X*	Insertion of TagBFP in *twk-18* at 5'
JIP1440-42	*twk-18(bln304) X*<br>*twk-18(bln305) X*<br>*twk-18(bln306) X*	Insertion of wrmScarlet in *twk-18* at 5'
JIP1328-9, JIP1331	*egl-23(bln269) IV*<br>*egl-23(bln270) IV*<br>*egl-23(bln272) IV*	Insertion of 2xMyc sequence in *egl-23* at 3'
JIP1253	*egl-23(bln252[Pmyo-2::mCherry::3'UTR unc-54])IV*	Deletion and replacement of *egl-23a* by *Pmyo-2::mCherry::3' UTR unc-54*

Supplementary Table 3: List of single strand oligonucleotides

Primer	Sequence (5' to 3')	Description
AF-ZF-827	CACTTGAACTTCAATACGGCAAGATGAGAATGA CTGGAACCGTACCGCATGCGGTGCCTATGGTAG CGGAGCTTCACATGGCTTCAGACCAACAGCCTAT	ssDNA repair template; *dpy-10(cn64)*
AF-JA-76	ATTTTGTGGTATAAAATAGCCGAGTTAGGAAAC AAATTTTTCTTTCAGGTTTCTCAGTAGTGACCA TGTGCGTGGATCTTGCGTCCACACATCTCAAGG CGTACTT	ssDNA repair template; *unc-58(e665)*
oSEM158	Gaaagcaagaaatcatcacatttgagtggctcat cgagaagagaaagcggtggaaaaGGAGGAGAACA AAAGTTGATTTCTGAAGAAGATTTGAACGGTGAA CAAAAGCTAATCTCCGAGGAAGACTTGtaattaa Aacttgtgtaattttaatgaaaataaagttttg tgctacgaattt	ssDNA repair template; insertion of 2xMyc sequence in *egl-23* at 3' *d10* site
oPT85	AACTACAGTATCCCAACTGATTGTGGTAACACATC ACGGCATGCACCACGGCTACCATAGGCACCACGAG CGGGATTGTGGCATGTGGTGTGTGTTCAGCGAGAG GCGGCGACACGTCGAGAG	ssDNA repair template; insertion of *d10 sequence in tag-68*
oSEM40	TGTGAGCTCAGCAGCTTCTCGTCGTTTCTTTTTTT GTATAAATTTGAAGAGCTACCATAGGCACCACGAG CGGACACTTATCGTCTGCACACTGACCTACCTTTT AGTTGGAGCAGCCGTATTT	ssDNA repair template; insertion of *d10 sequence in sup-9*
oCL60	CCGGAAAGCAAGAAATCATCACATTTGAGTGGCTC ATCGAGAAGAGAAAGCGGGCTACCATAGGCACCAC GAGCGGTTAAAACTTGTGTAATTTTTAATGAAAAT AAAGTTTTGTGCTACGAATTT	ssDNA repair template; insertion of *d10 sequence in egl-23* at 3' *d10* site
oCL58	TCTAAACATTTTCATTCTTCTCATTCCGTCACCCC ATTCTGTTCCCGGAATCGCTACCATAGGCACCACG AGCGGGAATCAAAAACTGCAATTTTCGAAAAGTAA GTTGGCCTTTTTGTGGGAAA	ssDNA repair template; insertion of *d10* sequence in *egl-23b*
oCL75	AGGTAGTACACGAAAGGTAGGAGGCAAGCGGGTGA CAAAAACAACCGGAAGCTACCATAGGCACCACGAG CGGGCGATTGTTGCGCAAGGAGTTTCTACGATTTT GACGACGTTTCAGAAAAC	ssDNA repair template; insertion of *d10* sequence in *twk-18*
oNZ7	caagagttggaccaatgggaatattggatgaagct tttggagatgagcctTACCGCTCGTGGTGCCTATG GTAGCgtcgatttttgattatcttgtagaatgctc gtgcaaattgtataattagt	ssDNA repair template; insertion of *d10* sequence in *twk-14*
oSEM171	gtgaatgatcacgcacgata	Primer pair for amplification of *d10* off-target site in *R12E2.15*
oSEM172	gtactgtggtggtggtggtg	
oCL77	CCAACCCCTCTCATCCTTTT	Primer pair for amplification of *d10* site in *twk-18(bln213)*
oCL78	TTTCATAGTCGATTTTCATTCAGA	
oPT95	GAAAATTTGGAACCGGGCTA	Primer pair for amplification of *d10* site in *tag-68(bln212)*
oTB586	CAACGTCTTCTGCATCGAAA	
oSEM31	ATGAACCTCCTAGTGCTCCG	Primer pair for amplification of *d10* site in *sup-9(bln226)*
oSEM32	GGAAATGGGCTCTCGTTGTG	
oCL44	AAAGCGTACAGCGAAGAAGC	Primer pair for amplification of *d10* site in *egl-23(bln220*
oCL34	TTCCAAGCATATTTGTGATCG	
oCL45	CGATTGTGAGCCAATGAGAA	Primer pair for amplification of *d10* site in *egl-23b(bln172)*
oCL46	CGCTTTTCAATTTTCCATGC	

oSEM140	GCCAAAAGAAGACCCATGAC	Primer pair for amplification of *d10* site in *twk-40(bln271)*
oSEM141	AAAAATCGCTCTAAATTTCCAGTT	

Supplementary Table 4: List of plasmids

Plasmid/ locus	Primer	Sequence (5' to 3')	Description
pSEM91/ *egl-23*	oCL101	ActcactatagggggcgcGcctcgacctgcaggtcgagctGGCTATATGGTTTGGAGGAA	LHR amplification
	oSEM275	TTTTCCACCGCTTTCTCTTC	
	oSEM276	AcatttgagtggctcatcgaGaagagaaagcggtggaaaaATGGTCAGCAAGGGAGAGGC	wrmScarlet amplification
	oSEM277	AaaactttattttcattaaaAattacacaagttttaattaCTTGTAGAGCTCGTCCATTC	
	oSEM278	TTAAAACTTGTGTAATTTTT	RHR amplification
	oCL106	TtcctggccttttgctggccttttgctcacatggcagCATGCAGTCGAGACATTTACATC	
pCL16/ *egl-23*	oCL101	ActcactatagggggcgcgcctcgacctgcaggtcgagctGGCTATATGGTTTGGAGGAA	LHR amplification
	oCL102	GcatgttctccttaatcagctcttcgcccttagacaccatTTTTCCACCGCTTTCTCTTC	
	oCL103	AcatttgagtggctcatcgagaagagaaagcggtggaaaaATGGTGTCTAAGGGCGAAGA	TagRFP-T amplification
	oCL104	CacaaaactttattttcattaaaaattacacaagttttaaTTAATTAAGTTTGTGCCCCA	
	oCL105	CtgcgacctccctagcaaactggggcacaaacttaattaaTTAAAACTTGTGTAATTTTTAATGAAAATAAAGTTTTGTGCTACGA	RHR amplification
	oCL106	ttcctggccttttgctggccttttgctcacatggcagCATGCAGTCGAGACATTTACATC	
pCL22/ *egl-23*	oCL101	ActcactatagggggcgcgcctcgacctgcaggtcgagctGGCTATATGGTTTGGAGGAA	LHR amplification
	oCL127	acaagcagttaactaggtgaaagtaggatgagacagcTTATTTTCCACCGCTTTCTCTTC	
	oCL128	tgagtggctcatcgagaagagaaagcggtggaaaataaGCTGTCTCATCCTACTTTCACC	SL2::TagRFP-T amplification
	oCL104	CacaaaactttattttcattaaaaattacacaagttttaaTTAATTAAGTTTGTGCCCCA	
	oCL105	CtgcgacctccctagcaaactggggcacaaacttaattaaTTAAAACTTGTGTAATTTTTAATGAAAATAAAGTTTTGTGCTACGA	RHR amplification
	oCL106	ttcctggccttttgctggccttttgctcacatggcagCATGCAGTCGAGACATTTACATC	
pSEM67/ *egl-23*	oCL101	actcactatagggggcgcgcctcgacctgcaggtcgagctGGCTATATGGTTTGGAGGAA	LHR amplification
	oSEM142	tgtacagtttcatatgcatattctccttaataagctctgatTTTCCACCGCTTTCTCTTC	
	oSEM143	TCAGAGCTTATTAAGGAGAA	TagBFP amplification
	oSEM146	aaaactttattttcattaaaaattacacaagttttaattaATTAAGCTTGTGACCCAGTT	
	oSEM145	CGCAAACGCCAAGACCACATATAGATCCAAGAAACCG	TagBFP2 point mutation
	oSEM144	TTTCTTGGATCTATATGTGGTCTTGGCGTTTGCGATGAGATGGCTACCGC	
	oSEM147	CtgcgacctcccgagcaaactgggtcacaagcttaattaatTAAAACTTGTGTAATTTTT	RHR amplification
	oCL106	TtcctggccttttgctggccttttgctcacatggcagCATGCAGTCGAGACATTTACATC	

pSEM69/ *egl-23*	oCL101	actcactatagggggcgcgcctcgacctgcaggtcgagctGGCTATATGGTTTGGAGGAA	LHR amplification
	oSEM142	tgtacagtttcatatgcatattctccttaataagctctgaTTTTCCACCGCTTTCTCTTC	
	oSEM143	TCAGAGCTTATTAAGGAGAA	TagBFP amplification
	oSEM146	aaaactttattttcattaaaaattacacaagttttaattaATTAAGCTTGTGACCCAGTT	
	oSEM147	ctgcgacctcccgagcaaactgggtcacaagcttaattaatTAAAACTTGTGTAATTTTT	RHR amplification
	oCL106	ttcctggccttttgctggccttttgctcacatggcagCATGCAGTCGAGACATTTACATC	
pSEM55/ *sup-9*	oSEM75	gactcactatagggggcgcgcctcgacctgcaggtcgagcGTGCAGCAGGAAGTGATGGA	LHR amplification
	oSEM74	TCTTCAAATTTATACAAAAAAAGAAACGACGAGAAGCTGCTGA	
	oSEM63	gcagcttctcgtcgtttcttttttgtataaatttgaagaATGGTGTCTAAGGGCGAAGA	TagRFP-T amplification
	oSEM72	tgcagacgataagtgacagggttctgatattttggcgcttATTAAGTTTGTGCCCCAGTT	
	oSEM77	AAGCGCCAAAATATCAGAACCC	RHR amplification
	oSEM76	cggttcctggccttttgctggccttttgctcacatggcagTAGTCATCCCGGAAAACGTC	
pSEM61/ *twk-18*	oCL117	gactcactatagggggcgcgcctcgacctgcaggtcgagcATGGGAATTGGTGCATTTTC	LHR amplification
	oSEM123	TTCCGGTTGTTTTTGTCACC	
	oSEM125	aaggtaggaggcaagcgggtgacaaaaacaaccggaaatgTCAGAGCTTATTAAGGAGAA	TagBFP amplification
	oSEM128	acgtcgtcaaaatcgtagaaactccttgcgcaacaatcgcATTAAGCTTGTGACCCAGTT	
	oSEM127	GCGATTGTTGCGCAAGGAGT	RHR amplification
	oCL120	cggttcctggccttttgctggccttttgctcacatggcagGTGAACAAGACCGCACAGAA	
pCL17/ *twk-18*	oCL117	gactcactatagggggcgcgcctcgacctgcaggtcgagcATGGGAATTGGTGCATTTTC	LHR amplification
	oCL118	gcatgttctccttaatcagctcttcgcccttagacaccatTTCCGGTTGTTTTTGTCACC	
	oCL91	cgaaaggtaggaggcaagcgggtgacaaaaacaaccggaaATGGTGTCTAAGGGCGAAGA	TagRFP-T amplification
	oCL92	acgtcgtcaaaatcgtagaaactccttgcgcaacaatcgcATTAAGTTTGTGCCCCAGTT	
	oCL119	actgcgacctccctagcaaactggggcacaaacttaatgcGATTGTTGCGCAAGGAGTTT	RHR amplification
	oCL120	cggttcctggccttttgctggccttttgctcacatggcagGTGAACAAGACCGCACAGAA	
pSEM87/ *twk-18*	oCL117	gactcactatagggggcgcgcctcgacctgcaggtcgagcATGGGAATTGGTGCATTTTC	LHR amplification
	oSEM123	TTCCGGTTGTTTTTGTCACC	
	oSEM127	GCGATTGTTGCGCAAGGAGT	RHR amplification
	oCL120	cggttcctggccttttgctggccttttgctcacatggcagGTGAACAAGACCGCACAGAA	
pSEM80/ *twk-40*	oSEM135	ccgccagatcttccggatggctcgagtttttcagcaagaTAAGGACGGTTGCAATTAATC	LHR amplification
	oSEM136	aataagctagcaccgctcgtggtgcctatggtagcaccggTTCAATTGAGGCCAATGCTC	
	oSEM137	attgaaccggtgctaccataggcaccacgagcggtgctagCTTATTTTTAGATTAATTGT	RHR amplification
	oSEM138	atggcagctgagaatattgtaggagatcttctagaaagaTTTTTG	

		GCGAAAAATTCAGGT	
	oMM7	gatttatcgattttggagcattggcctcaattgaaagttggcaatcgctatggtcaattctcactggaagaactcaagaagttcatgttatggtgtctaagggcgaagagctgattaagg	TagRFP-T amplification
	oTB600	acacccgttaaaacaaaaaaaaaacaaacacaattaatctaaaaataaggatccgccactacctccagagccaccATTAAGTTTGTGCCCCAGTTTGC	
	oTB601	cacaaacttaatggtggctctggaggtagtggcgGAACAGAATACAAAACGCGACTTTGTGATGCGTTCCGCCGTGAAGGATACTGCCCGTACAACGACAATTGCACATATGCTCACGGACAAGATGAGCTGAGAGTTCCGTAActtatttttagattaattgtgtttgttttttttttgttttaacgggtg	Integration of ZF1 sequence
pPT46/ *tag-68*	oPT86	aattgcaaatctaaatgtttCCACATGCCACAATCCATCGgttttagagctagaaatagc	Forward primer
	oPT87	gctatttctagctctaaaacCGATGGATTGTGGCATGTGGaaacatttagatttgcaatt	Reverse primer
pPT47/ *tag-68*	oPT88	gctatttctagctctaaaacCAATCCATCGTGGTGCATGCaaacatttagatttgcaatt	Forward primer
	oPT89	gctatttctagctctaaaacCAATCCATCGTGGTGCATGCaaacatttagatttgcaatt	Reverse primer
pSEM46/ *sup-9*	oSEM27	aatgcaaatctaaatgtttCCACATGCCACAATCCATCGgttttagagctagaaatagc	Forward primer
	oSEM28	gctatttctagctctaaaacAAATATTAAGAAGAAGCTGCaaacatttagatttgcaatt	Reverse primer
pSEM48/ *sup-9*	oSEM43	aattgcaaatctaaatgtttGTGTGCAGACGATAAGTGACgttttagagctagaaatagc	Forward primer
	oSEM44	gctatttctagctctaaaacGTCACTTATCGTCTGCACACaaacatttagatttgcaatt	Reverse primer
pSEM50/ *sup-9*	oSEM47	aattgcaaatctaaatgtttGTGTGCAGACGATAAGTGACAgttttagagctagaaatag	Forward primer
	oSEM48	ctatttctagctctaaaacTGTCACTTATCGTCTGCACACaaacatttagatttgcaatt	Reverse primer
pCL11/ *egl-23*	oCL67	attgcaaatctaaatgtttGTCATCGAGAAGAGAAAGCGGgttttagagctagaaatagc	Forward primer
	oCL68	gctatttctagctctaaaacCCGCTTTCTCTTCTCGATGACaaacatttagatttgcaat	Reverse primer
pCL8/ *egl-23b*	oCL61	attgcaaatctaaatgtttgCCATTCTGTTCCCGGAATCagttttagagctagaaatagc	Forward primer
	oCL62	gctatttctagctctaaaactGATTCCGGGAACAGAATGGcaaacatttagatttgcaat	Reverse primer
pCL9/ *egl-23b*	oCL63	attgcaaatctaaatgtttGAGTTTTTGATTCCATGATTCgttttagagctagaaatagc	Forward primer
	oCL64	gctatttctagctctaaaacGAATCATGGAATCAAAAACTCaaacatttagatttgcaat	Reverse primer
pCL12/ *twk-18*	oCL69	aattgcaaatctaaatgtttGTGACAAAAACAACCGGAAAgttttagagctagaaatagc	Forward primer
	oCL70	gctatttctagctctaaaacTTTCCGGTTGTTTTTGTCACaaacatttagatttgcaatt	Reverse primer
pCL13/ *twk-18*	oCL71	attgcaaatctaaatgtttgTGCGCAACAATCGCCATTTCgttttagagctagaaatagc	Forward primer
	oCL72	gctatttctagctctaaaacGAAATGGCGATTGTTGCGCAcaaacatttagatttgcaat	Reverse primer
pMM1/ *twk-40*	oMM1	AATTGCAAATCTAAATGTTTgcattggcctcaattgaaagtGTTTTAGAGCTAGAAATAGC	Forward primer
	oMM2	GCTATTTCTAGCTCTAAAACACTTTCAATTGAGGCCAATGCAAACATTTAGATTTGCAATT	Reverse primer
pNZ1/	oNZ1	ATTGCAAATCTAAATGTTTGcctttaatcagagttttactGTTTT	Forward primer

twk-14		AGAGCTAGAAATAGC	
	oNZ2	GCTATTTCTAGCTCTAAAACagtaaaactctgattaaaggCAAACATTTAGATTTGCAAT	Reverse primer
pNZ2/ *twk-14*	oNZ3	ATTGCAAATCTAAATGTTTgaaactctgattaaaggtttaGTTTTAGAGCTAGAAATAGC	Forward primer
	oNZ4	GCTATTTCTAGCTCTAAAACtaaacctttaatcagagtttcAAACATTTAGATTTGCAAT	Reverse primer
pNZ3/ *twk-14*	oNZ5	ATTGCAAATCTAAATGTTTgccgagtaaaactctgattaaGTTTTAGAGCTAGAAATAGC	Forward primer
	oNZ6	GCTATTTCTAGCTCTAAAACttaatcagagttttactcggcAAACATTTAGATTTGCAAT	Reverse primer

Supplementary Methods

Building sgRNA expression vectors using pPT2

This protocol describes the steps and tools used to generate sgRNA expression vectors using the pPT2 vector backbone. See the materials and methods section for the required reagents (e.g. Gibson assembly reagents, sequencing primers).
We recommend the online service benchling.com for sgRNA, oligo and vector design.

I. Identifying a suitable protospacer motif

• A protospacer is a 19-20 bp sequence flanked at its 3' end by an NGG PAM (protospacer adjacent motif). Different online tools are available to identify possible protospacers in a region of interest (crispr.mit.edu ; tefor.net/crispor/crispor.cgi ; benchling.com).

• When multiple protospacer sequences are possible, select the closest (to the site to engineer) and/or the most specific sequence (use the off-target prediction tool provided by benchling for example). In general, four non-matching bases should be enough to significantly reduce off-target cutting, especially if the mismatches are located in the 3′ region of the protospacer (Hsu *et al.* 2013).

II. Building the sgRNA vector sequence *in silico*

• The pPT2 vector contains the U6 promoter and 3′ UTR of *K09B11.12* (Friedland *et al.* 2013) and two restriction sites (PmeI and SexAI) to linearize the vector, followed by the invariant sgRNA scaffold sequence (see Figure 1A).
• To generate the sgRNA expression vector sequence, insert the protospacer sequence (**without** the PAM, i.e. NGG) between the U6 promoter and the sgRNA scaffold as shown in figure 1B.
• If the selected protospacer sequence does not begin with a guanine residue, add this nucleotide manually to the 5′ of the protospacer (i.e. resulting in a ″19+1″ bp insertion in pPT2, see figure 1B).
• Name this vector pXYn where XY are the initials of the person building the vector and n the number of the vector. Accordingly, the protospacer sequence is then labeled CRpXYn (generate a "feature" with the sequence, excluding the added G, to identify it easily in the genomic sequence).
• Generate one 60 bp oligonucleotide centered on the protospacer sequence as shown in figure 1C (forward or reverse). **Gibson assembly can be performed with a single primer** (see IIIa). Alternatively, generate two complementary 60 bp oligonucleotides centered on the protospacer sequence as shown in figure 1C (see IIIb).

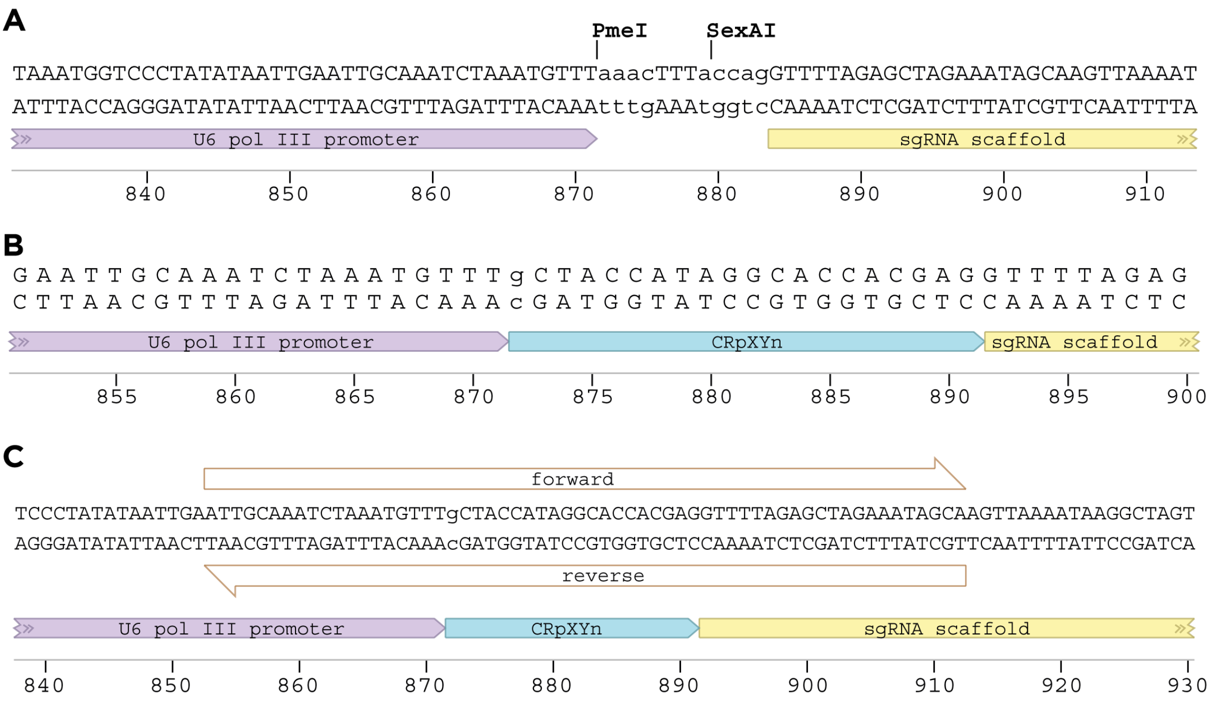

Figure 1 - Insertion of a protospacer sequence into the pPT2 sgRNA expression vector.

IIIa. Building the sgRNA vector using a single oligonucleotide

The protospacer sequence is incorporated into the pPT2 vector as follows:

1 | Linearize pPT2 using the *PmeI* and *SexAI* restriction enzymes.

2 | Purify the linearized pPT2 vector using gel extraction.

3 | **Gibson assembly**
• Thaw an aliquot of Gibson Master Mix and keep on ice.
• Mix 100 ng of linearized pPT2 vector with 1 μL of 100 μM (or 0.1 nmole) of single strand oligonucleotide and add water up to 5 μL if necessary.
• Add 15 μL of Gibson Master Mix to the DNA mix.
• Incubate at 50°C for 15 to 60 minutes (60 minutes is optimum).
• Transform 5 μL of this reaction, and grow on LB+Ampicilin plates.
• Perform a control experiment (Gibson assembly without oligonucleotide) for each new batch of linearized pPT2 vector.

4 | **Sequence validation**
• Due to the high efficiency/specificity of Gibson assembly, colony PCR is not required.
• Validate the resulting vector by sequencing with pJET1.2fwd or pJET1.2rev.

IIIb. Building the sgRNA vector using oligonucleotide dimers

• The protospacer sequence is incorporated into the pPT2 vector as follows.

1 | Linearize pPT2 using the *PmeI* and *SexAI* restriction enzymes.

2 | Purify the linearized pPT2 vector using gel extraction.

3 | **Hybridize oligonucleotides** (using a thermocycler)
• Add 1 µL of each oligonucleotide (at 100 µM) to 18 µL of water.
• Run the program below on a thermal cycler to anneal primers.
• Add 30 µL of water to the resulting sample.

95°C	10 min
95°C to 85°C	[-2.0 °C/s]
85°C	1 min
85°C to 75°C	[-0.3°C/s]
75°C	1 min
75°C to 65°C	[-0.3°C/s]
65°C	1 min
65°C to 55°C	[-0.3°C/s]
55°C	1 min
55°C to 45°C	[-0.3°C/s]
45°C	1 min
45°C to 35°C	[-0.3°C/s]
35°C	1 min
35°C to 25°C	[-0.3°C/s]
25°C	1 min
4°C	Hold.

4 | **Gibson assembly**
• Thaw an aliquot of Gibson Master Mix and keep on ice.
• Mix 100 ng of linearized pPT2 vector with 1 µL of hybridized oligonucleotides and add water up to 5 µL if necessary.
• Add 15 µL of Gibson Master Mix to the DNA mix.
• Incubate at 50°C for 15 to 60 minutes (60 minutes is optimum).
• Transform 5 µL of this reaction, and grow on LB+Ampicilin plates.
• Perform a control experiment (Gibson assembly without oligonucleotide dimer) for each new batch of linearized pPT2 vector.

5 | **Sequence validation**
• Due to the high efficiency/specificity of Gibson assembly, colony PCR is not required.
• Validate the resulting vector by sequencing with pJET1.2fwd or pJET1.2rev.

Materials and Methods

pPT2 sequence file

The annotated sequence file for the pPT2 vector can be downloaded as a Genbank format (readable in ApE and benchling.com) at the following link:

http://www.excitingworms.eu/resources/pPT2.gb

Homemade Gibson Assembly Reagents

Based on *Methods in Enzymology, Volume 498*
CHAPTER FIFTEEN part 5: Enzymatic Assembly of Overlapping DNA Fragments (Daniel G. Gibson)

5X ISO Buffer, 2 mL (store at -20°C)

Tris-HCl pH=7,5 1M	1 mL
$MgCl_2$ 2 M	50 μL
dGTP 100 mM	20 μL
dATP 100 mM	20 μL
dTTP 100 mM	20 μL
dCTP 100 mM	20 μL
DTT 1 M	100 μL
NAD^+ 50 mM	200 μL
PEG8000	0.5 g
H_2O	add up to 2 mL

• Prepare 320 μL aliquots and store at -20°C.

Gibson Master Mix, 1.2 mL (store at -20°C)
For the assembly of DNA molecules with overlaps of 20-80 bp.

5X ISO buffer	320 μL
T5 Exonuclease (10 U/μL)	0.64 μL
Phusion (2 U/μL)	20 μL
Taq Ligase (40 U/μL)	160 μL
H_2O	700 μL

Note: For overlaps that are larger than 80 bp, 3.2 μL exonuclease is used in this mix.

• Separate into 50 μL aliquots. Store at -20°C. The enzymes remain active after 10 cycles of freeze-thaw.

Sequencing Primers

pJET1.2fwd 5′-cgactcactatagggagagcggc-3′
pJET1.2rev 5′-aagaacatcgattttccatggcag-3′
